# Exploring 3D anatomy: A free online course supporting novice learner inquiry and spatial conceptualization

**DOI:** 10.1002/ase.70288

**Published:** 2026-06-11

**Authors:** Jiayi Zhang, Moksh Sachdeva, Calum McCaig, Eleanor Gordon, Leonard Shapiro, Iain D. Keenan

**Affiliations:** ^1^ School of Medicine, Faculty of Medical Sciences Newcastle University Newcastle upon Tyne UK; ^2^ The James Cook University Hospital Middlesbrough UK; ^3^ Learning and Teaching Development Service Newcastle University Newcastle upon Tyne UK; ^4^ The Department of Family, Community and Emergency Care, Primary Health Care Team, Faculty of Health Sciences University of Cape Town Cape Town South Africa

**Keywords:** anatomical illustration, anatomy education, anatomy teaching, digital anatomy, gross anatomy, topographic anatomy

## Abstract

Application of three‐dimensional anatomical knowledge is essential for patient examination, diagnosis, and treatment and is crucial in the training of medical and healthcare students. Moreover, critical observation and spatial awareness are vital for learner understanding of three‐dimensional anatomical structures and relationships. However, there are logistical barriers to curricular integration of teaching that explicitly targets these skills. *Exploring 3D Anatomy* is an open online course, collaboratively produced at two universities in the United Kingdom and South Africa, and available free of charge to institutional and non‐institutional users via Canvas (Instructure Europe, London, UK). The course is designed to develop three‐dimensional spatial awareness remotely, using accessible household objects and art‐based learning. *Exploring 3D Anatomy* is underpinned by pedagogical principles including multimodal, experiential, and social learning; critical observation and visualization; and evidence‐informed art‐based learning approaches including *Haptico‐visual observation and drawing* and *Observe‐reflect‐draw‐edit‐repeat*. A phenomenological study was implemented to investigate learner perceptions of the pedagogical, social, and practical elements of *Exploring 3D Anatomy*. Undergraduate medical students (*n* = 8) at a United Kingdom medical school participated in two focus groups. Combined themes were constructed using double‐coding and interpretative phenomenological analysis. A nested set of four themes was produced, each comprising a dichotomy or spectrum of views between opposing sub‐themes: Inquiry (Strategic–Exploratory); Dialogue (Knowledge–Understanding); Cognition (Compartmental–Conceptual); Experience (Novice–Advanced). This work provides insights and implications for educators seeking to provide training in important but neglected skills beyond the boundaries of formal curricula, through implementation of flexible, accessible, and remote learning and training strategies in anatomical education.

## INTRODUCTION

Within the modern post‐pandemic context of anatomical education, degree program curricula are under increased time and workload pressures, combined with increasing student numbers and financial constraints to deliver high‐quality education that focuses on stakeholder and regulator determined specific anatomical knowledge content.^1^, ^2^, ^3^, ^4^ As such, there is little time to focus on explicit training in cognitive skills that can constitute crucial fundamental underpinnings that provide mental frameworks upon which novice learners can build their foundational understanding of anatomy. To address the development of such skills, art‐based learning approaches, particularly those involving drawing, have been extensively implemented into anatomy teaching and are prevalent within the anatomical and broader educational literature.^5^, ^6^, ^7^, ^8^, ^9^, ^10^ Such drawing approaches also include *Observe‐reflect‐draw‐edit‐repeat*,^11^ *Haptico‐visual observation and drawing*,^12^, ^13^ *Haptic surface painting*,^14^ and drawing on three‐dimensional printed models,^15^ all previously described and reported by the authors. Because of their focus on observation and visualization of the three‐dimensional form of anatomical features, art‐based approaches are likely to be valuable for enhancing spatial learning.^16^, ^17^, ^18^, ^19^, ^20^, ^21^, ^22^

### Visual and haptic spatial awareness in anatomical education

Observing and comprehending the arrangement of anatomical structures in three‐dimensional space within the human body is essential for learning and practice in medicine, medical sciences, and healthcare professions.^23^, ^24^, ^25^ Because objects have both internal volume and external form, *spatial awareness* (also known as visual–spatial thinking) can be defined as an integral understanding of three‐dimensional space and structure,^16^, ^25^ both cognitively through visualization and in the physical world through observation (Table 1). In turn, *spatial ability* can be defined as the application of spatial awareness when performing specific spatial tasks such as cognitive visualization and mental manipulation of three‐dimensional objects and cross‐sectional images^16^, ^26^, ^27^, ^28^, ^29^ (Table 1). Consequently, effective spatial awareness and spatial ability are likely to be crucial for anatomy learning with three‐dimensional and cross‐sectional resources (*spatial understanding*) and in clinical practice, surgery, and diagnostic image interpretation (*spatial skills*) (Table 1). Fortunately, spatial awareness may be improved through the practice of specific *spatial training* exercises^12^, ^13^, ^16^ (Table 1).

**TABLE 1:** Terminology as defined for the purposes of this study.

Term	Definition
Cognitive spatial awareness	Cognitive comprehension of general principles of three‐dimensional space and structure achieved through visualization
Physical spatial awareness	Comprehension of general principles of three‐dimensional physical objects in three‐dimensional physical space achieved through observation
Spatial ability	Application of *spatial awareness* to specific cognitive tasks, e.g. visualization, mental manipulation
Spatial understanding	Application of *spatial awareness* and/or *spatial ability* to comprehension of discipline‐specific knowledge or concepts, e.g., anatomical size, shape, position, relationships
Spatial skill	Application of *spatial awareness* and/or *spatial ability* to manual or cognitive tasks, e.g., patient examination, surgery, diagnostic image interpretation
Spatial training	Practical exercises or activities that explicitly or implicitly develop *spatial awareness*, *spatial ability*, *spatial understanding*, and/or *spatial skills*

*Note*: An individual learner may be placed along a spectrum in terms of their relative levels of *cognitive* and *physical spatial awareness*, *spatial ability*, *spatial understanding*, or *spatial skills*, which are likely to vary over time and in different contexts, and may be influenced by their exposure to *spatial training*.

While spatial ability and spatial training may not directly influence anatomy knowledge test scores,^18^, ^30^, ^31^ this is likely because three‐dimensional spatial anatomy is not typically or explicitly tested in assessments.^22^ This idea is supported by studies investigating spatial anatomy performance following three‐dimensional learning activities and spatial training, with statistically significant improvements reported.^26^, ^32^, ^33^, ^34^, ^35^, ^36^, ^37^ There also appears to be a reciprocal relationship at play, with anatomical dissection being shown to improve spatial ability,^38^ and with spatial awareness and spatial ability enhancing surgical skills.^39^

Furthermore, recent work has demonstrated that haptic observation skills correlate with visual spatial ability^19^, ^40^; that haptic perception of anatomy can supplement visual skills in individuals with low spatial ability^41^; and that hand gesture‐based teaching can reduce cognitive load, support construction of cognitive models, and enhance recall and application of spatial anatomy knowledge.^42^, ^43^ These findings suggest that a combination of visual and haptic modalities in practical and remote learning environments is likely to provide greater benefits to anatomy learners beyond visual‐only methods. It is therefore recommended that educators seeking to enhance three‐dimensional spatial understanding in their learners should include implicit spatial training activities in their teaching that combine multimodal visual and physical resources^15^, ^26^ and explicit visual and haptic spatial training, such as the *Haptico‐visual observation and drawing* method,^12^, ^13^, ^16^ and *Haptic surface painting*.^14^

### Online learning courses

The challenges of incorporating in‐person art‐based multimodal spatial awareness training into anatomy curricula have been previously reported by the authors.^13^ The authors described plans for an open online course to expand the boundaries of delivery to wider learner populations; to bring together the lead course educators (L.S. and I.D.K.) who are based on different continents and hemispheres, and subsequently, to address the heightened need for remote delivery due to the COVID‐19 pandemic.^13^, ^44^

Consequently, the authors have designed and created an open online course *Exploring 3D Anatomy*, which is based on the principles described above and delivered via the Instructure Canvas (Instructure Global, London, UK) learning management system. The course is intended to develop the spatial awareness of learners for application to crucial three‐dimensional and cross‐sectional anatomical concepts. Based on defined online course taxonomies,^45^, ^46^ *Exploring 3D Anatomy* can be described as a small‐scale, open, free, formative, instructor‐guided, vocational open online course. The course was initially launched internally on the Newcastle University Canvas learning environment during academic year 2021/22 for students registered at Newcastle University, UK and University of Cape Town, South Africa. An international launch via the external‐facing Instructure platform has provided open access to any user globally and coincided with World Anatomy Day, October 15, 2023. The external Instructure course has achieved a total of 460 users (2023–2026), while 4447 users have joined the identical internal Canvas version since launch (2021–2026).

While open online courses may no longer be at the height of their popularity, notwithstanding limitations in participant attrition and engagement,^47^, ^48^ and although significant learning gains may not yet have been widely demonstrated,^49^, ^50^ online anatomical and healthcare education courses can serve an important function. When implemented effectively and aimed at specific audiences, these courses can deliver training for continuing professional development, lifelong learning, and transferable skills, that are of wide applicability to learners and serve as effective supplements to (rather than replacements for) conventional teaching, in areas that may be under‐represented in formal curricula.^50^, ^51^, ^52^, ^53^, ^54^ Increased learner interest, motivation,^52^ skills development,^55^ knowledge enhancements,^56^ and long‐term retention^57^ have been reported, and online learners have benefited from communications with international peers within course discussion forums.^58^

### Conceptual framework

The *Exploring 3D Anatomy* course is based on a previously proposed flexible self‐directed learning approach.^13^ This course includes text‐based guidance in observation, practical activities, and reflections around short videos hosted and narrated by authors L.S. and I.D.K. The modular course structure is deliberately underpinned by *Observe‐reflect‐draw‐edit‐repeat* experiential learning cycles^11^ and consists of multiple learning exercises (Figure 1) based on the principles of *Haptico‐visual observation and drawing*.^12^, ^13^ Each exercise requires learners to engage in haptic and visual observation during practical art‐based activities using easily available and accessible household objects (Figure 2). Within each exercise, learners observe a video demonstration of (and sometimes simultaneously carry out) a practical learning activity, reflect on the activity and their understanding of it, and carry out the activity themselves or practice it again. Having done so, learners are encouraged to further reflect on their experiences and what they have learned from the activity, in terms of development and modification of their actions and understanding. Learners then repeat this process in further activities that are designed to gradually build their learning and skills across consecutive exercises. *Exploring 3D Anatomy* also aims to engage learners in peer‐peer and peer‐educator interactions within open discussion boards, in order to recreate the collaborative learning environment of in‐person *Haptico‐visual observation and drawing* workshops.^13^ The elements of building upon prior knowledge, experiential cycles, and social learning found within the *Exploring 3D Anatomy* course are underpinned by theories of cognitive constructivism,^59^ experiential learning,^60^ and social learning^61^ respectively.

**FIGURE 1:**
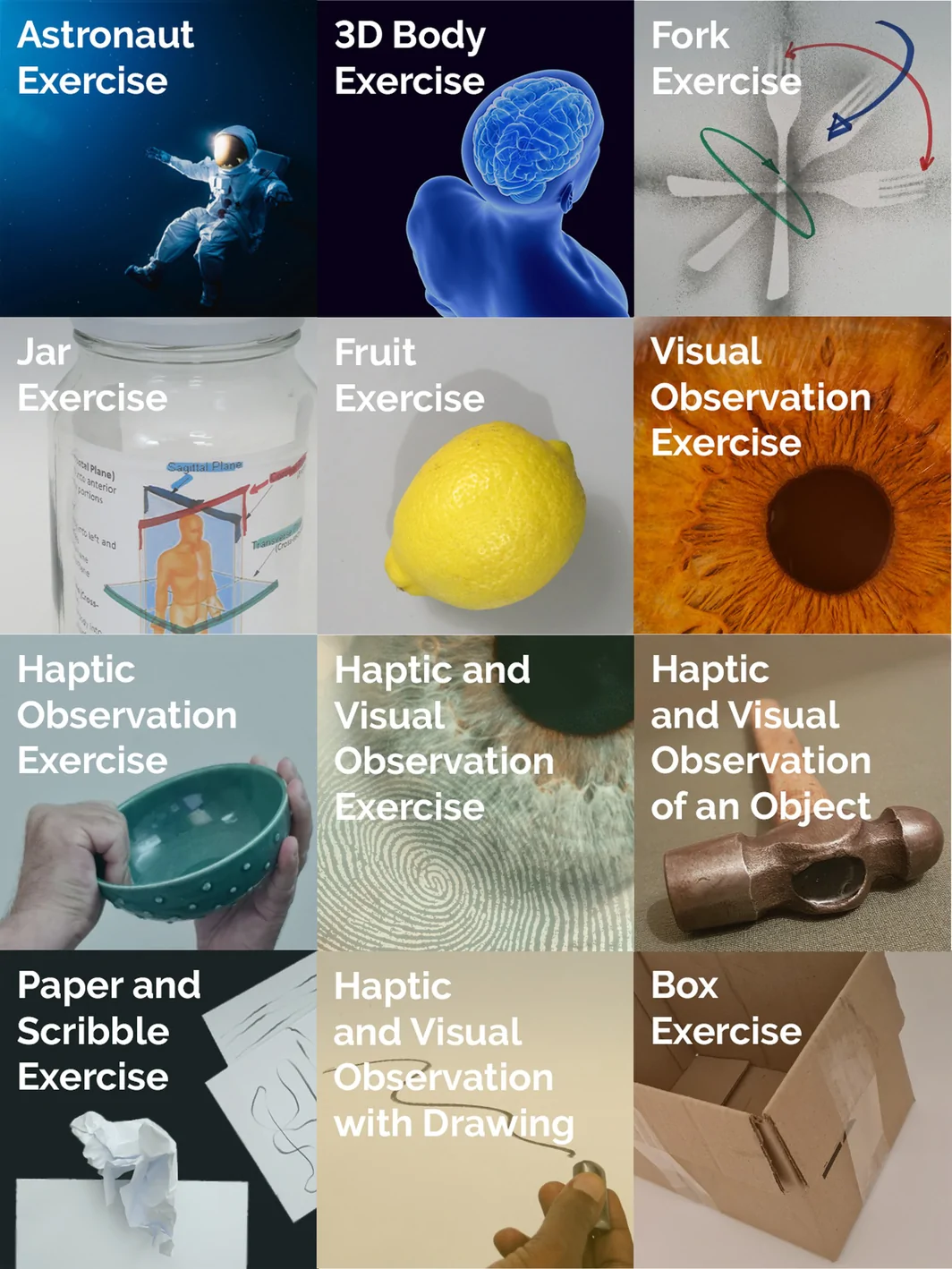
Montage of image tiles with links to course content presented to learners on the *Exploring 3D Anatomy* homepage for information and navigation. The course is structured in distinct modular exercises to enable learners to move through tasks in sequence, beginning with the *Astronaut Exercise*. Each module is unlocked as learners move through the course in the recommended sequence (left to right; top to bottom) and can be revisited at any time.

**FIGURE 2:**
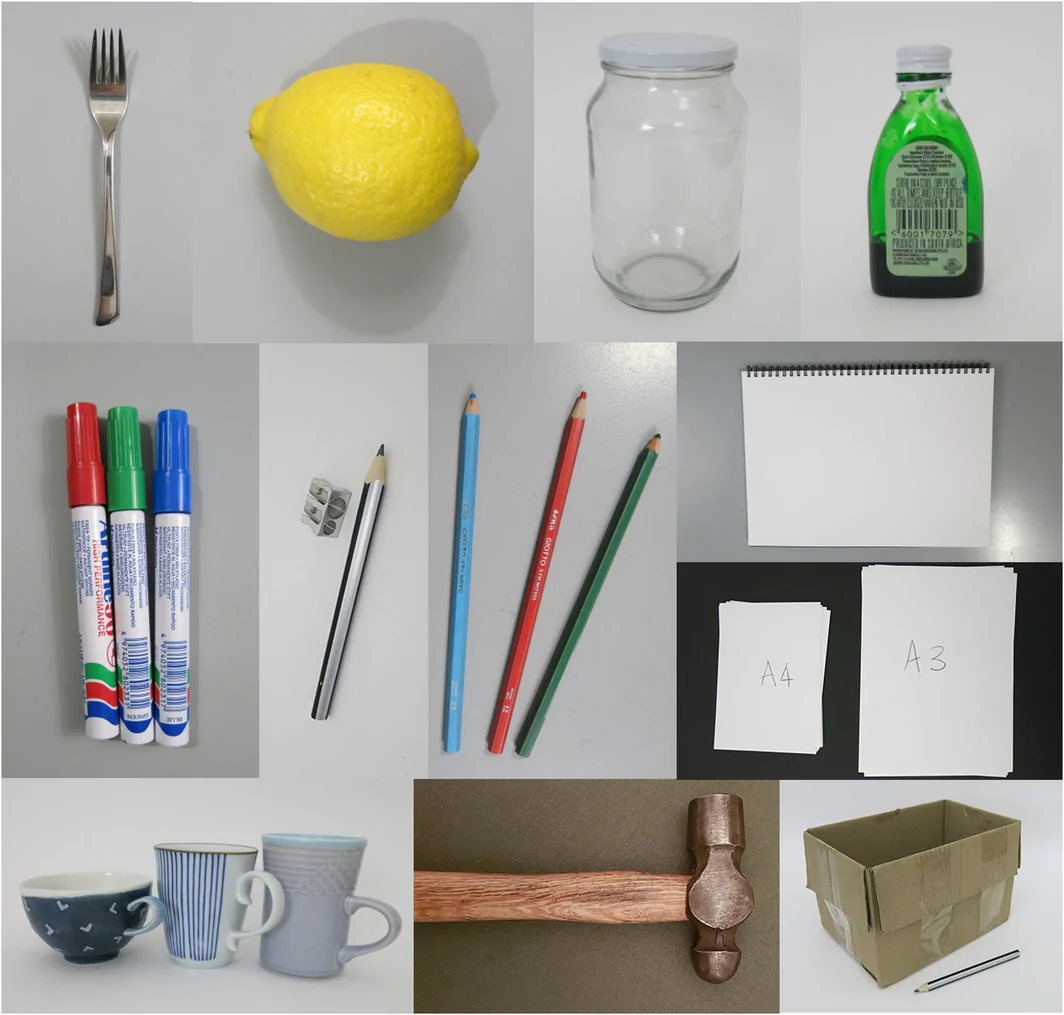
Montage of exemplar household objects and accessible materials required for *Exploring 3D Anatomy* exercises (left to right; top to bottom: Fork, lemon, glass jar, food dye, colored permanent markers, pencil and sharpener; colored pencils, notepad, A4 (letter size) and A3 (tabloid/ledger size) paper, cups/mugs, ballpeen hammer, cardboard box. Anatomical resources/models are not required nor recommended.

Having designed this course for anatomy learners, it was crucial to investigate their experiences of anatomy learning and *Exploring 3D Anatomy*, their perceptions of course activities with respect to the development of their spatial awareness, and their views regarding the application of the course to an understanding of three‐dimensional anatomy.

## STUDY GOALS

### Aim

This study aimed to explore and understand medical student perspectives and experiences of *Exploring 3D Anatomy* in the context of a United Kingdom medical school, in order to investigate the value of the course as a supplement to curricular anatomical education and to provide insights for future development.

### Research questions

1. How do medical students experience anatomy learning?
2. How do medical students experience the *Exploring 3D Anatomy* course and learning exercises?
3. How do medical students experience *Exploring 3D Anatomy* in the context of their anatomy education?

To answer these questions, a phenomenological methodology utilizing focus group methods and interpretative phenomenological analysis was implemented within an interpretivist epistemological paradigm, in which the collection of subjective learner perceptions is implicit and essential.^62^, ^63^ Based on the nature of the course and the findings of previous work,^13^, ^51^, ^52^, ^54^, ^55^, ^56^, ^57^, ^58^ it was expected that learners would discuss their experiences of *Exploring 3D Anatomy* as a supplementary learning resource, their spatial awareness of three‐dimensional and cross‐sectional anatomy, and their interactions with other students and educators within the course.

## METHODS

### Educational context

The format and delivery of integrated anatomical learning and teaching within the post‐2017 case‐based curriculum of the undergraduate Bachelor of Medicine and Surgery (M.B.B.S.) medical program at Newcastle University School of Medicine has previously been described by the authors.^3^, ^28^, ^29^, ^64^, ^65^ Anatomy is delivered within the preclinical years 1 and 2 of the M.B.B.S. program, which runs between late September and early May. The course begins with an induction week and a two‐week foundational “*Genes to Function to Impact*” unit in basic medical sciences, which includes an “*Introduction to Anatomy*” lecture covering basic structures, functions and terminology, and a practical “*Anatomy Induction*” session to provide students with their first opportunity to view and handle donor specimens in the laboratory. The foundational unit is followed by 25 case‐based units across the preclinical curriculum, 15 of which include synchronous lecture, practical, and seminar‐based anatomy teaching, and asynchronous self‐directed study. *Exploring 3D Anatomy* was originally launched and publicized to preclinical Newcastle MBBS students in October 2021. From September 2023, new Year 1 students entering the MBBS program attend an in‐person seminar for formal training in digital anatomy resources including *Exploring 3D Anatomy*.

### Exploring 3D anatomy

#### Course development

While the majority of video production for *Exploring 3D Anatomy* was achieved via videoconferencing software (Zoom Video Communications, San Jose, California, USA) in summer 2021 during the Covid‐19 pandemic, remote filming of content would have in any case been required due the international locations of creators I.K. and L.S. in the United Kingdom and South Africa, respectively. The Newcastle University Faculty of Medical Sciences Technology‐Enhanced Learning team primarily utilized the technological pedagogical content knowledge (TPACK) approach^66^, ^67^ to underpin the technological and pedagogical elements of course design. As *Exploring 3D Anatomy* was initially adapted from art‐based teaching^11^ and workshop^13^ sessions, it was desirable to recreate this experience within the course. A semi‐scripted and informal approach to production enabled flexibility and creativity in the design and recording of exercises and resulted in authentic lecture‐style demonstrations and conversations between the educators, which would not have been possible in a fully scripted studio environment. This expert‐learner discourse format was developed to closely resemble an in‐person environment, which could ease learners into unfamiliar art‐based learning activities. Video editing was therefore performed with the intention of preserving natural conversational flow, to encourage learners in their flexible exploration of three‐dimensional anatomy and to enable the educators to become embodied within the course. It was anticipated that learners would then achieve greater connection to L.S. and I.K., which would in turn encourage their participation in activities and discussions, and would therefore enhance their learning experience. Occasional video re‐shoots were required to optimize clarity and delivery, and the professional appearance of videos was achieved with postproduction editing and visual and quality enhancements, performed by learning technologists and content developers from the Technology‐Enhanced Learning team.

#### Course structure

In addition to the creation of the individual video resources, the webpages in which these videos are embedded have been deliberately designed to provide clear instructions. Guidance through a sequence of tasks and the gathering of appropriate materials, and the setting of expectations (e.g., the time required to complete tasks) are all included. Each page is arranged in the required order within a modular format, which allows learners to navigate back and forward through consecutive tasks. A general “Course Information” module introduces the learners to the educators, and includes course aims, required materials, and an explanation of how to use and navigate the course both pedagogically and logistically. Learners are encouraged to read this supporting information before starting on course exercises and are provided with information on how to find help if needed.

While these terms have broader educational definitions, within the context of *Exploring 3D Anatomy*, “exercise” here refers to the entirety of an individual course module (Figure 1), while “activity” refers to the specific tasks and actions carried out by learners when studying each exercise. Distinct *Exploring 3D Anatomy* exercises support learner progression from a simple starting point of cognitive visualization activities through visual and haptic activities involving household objects (Figure 2). Drawing activities are included at deliberate points in the course, with marks made on paper by learners serving as visual representations of their cognitive understanding.^13^ To encourage development of an online community of social learning,^68^ individual learners are invited to consider their own experiences of each *Exploring 3D Anatomy* exercise and to provide written reflections within the online discussion board for each activity. Educator reflection videos are also included in each exercise to further support an environment of reassurance and inspiration to inexperienced learners. Learners are encouraged to read and respond to discussion board reflections in order to learn from each other, while educators monitor threads and provide encouragement and supportive feedback. For each exercise, an anticipated approximate total time that learners will spend on each exercise is provided below. This includes the expected time for reading text‐based guidance, observing video demonstrations, carrying out activities, and reflecting on and discussing their experiences.

##### Astronaut exercise (<10 min)

*Exploring 3D Anatomy* begins with a cognitive visualization exercise, which requires the learner to use their imagination to create mental images. In this exercise, the learner follows an embedded video, which includes audio narration by educator L.S. and a sequence of digitally produced still images of an astronaut floating in space (Figure 3). The astronaut is shown from several different visual perspectives, to indicate that they are floating in space and rotating around their own axis. After learners have viewed this sequence, the narrator asks learners to close their eyes and visualize a mental picture of the astronaut floating in space, and then to visualize themselves as the rotating astronaut. As is the case for each activity and exercise, learners are invited to contribute to the *Astronaut Exercise* discussion boards to describe their reflections.

**FIGURE 3:**
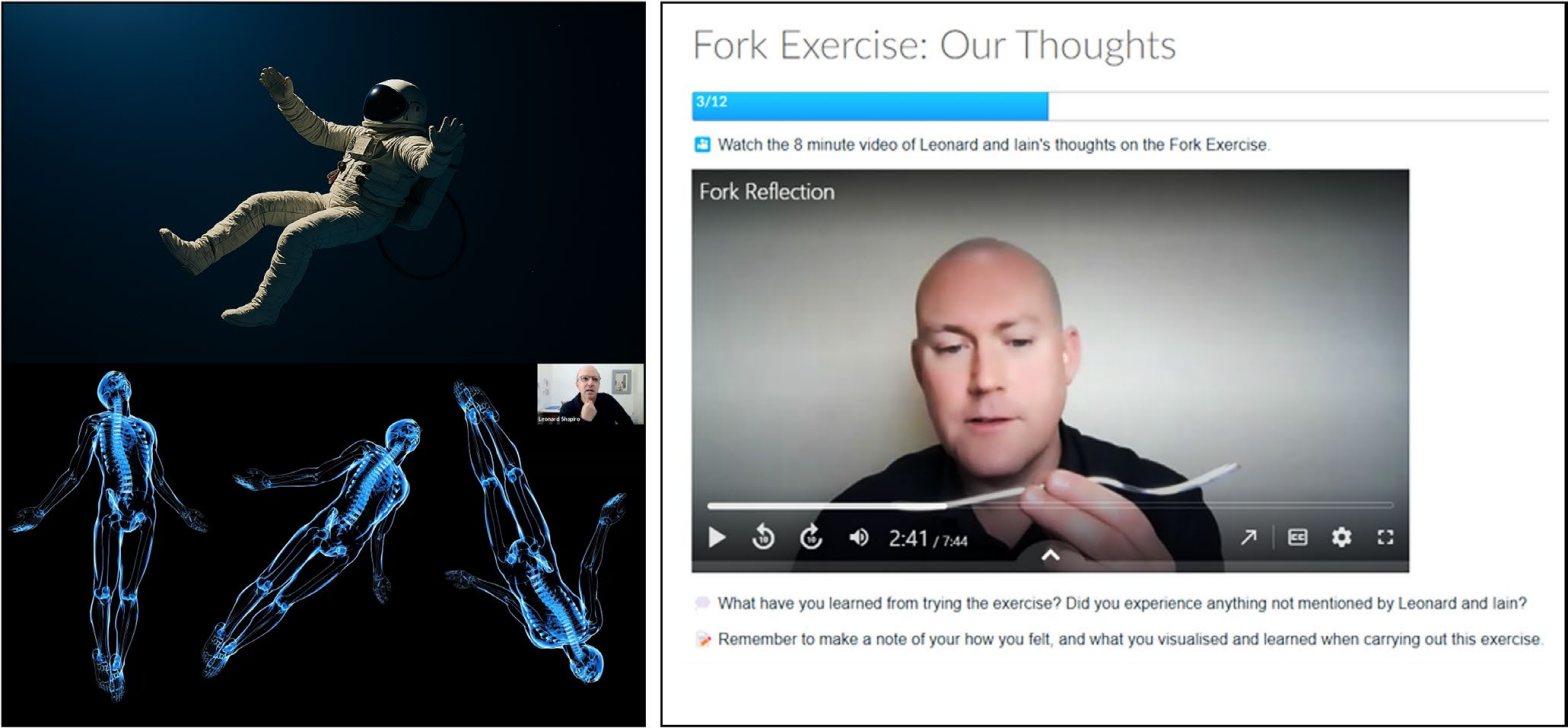
Examples of still image of an astronaut and (top left) and transparent three‐dimensional representations of the human body (bottom left) (image usage license purchased from 123RF) from embedded *Astronaut Exercise* and *3D Body Exercise* videos. Screenshot of *Exploring 3D Anatomy* platform showing blue course progress bar (top right); embedded *Fork Exercise* video showing I.K. demonstrating spatial manipulation, and haptic and visual observation of a simple three‐dimensional household object (center right); and text‐based guidance and prompts for learners to reflect on their own experiences of the activity (bottom right).

##### 3D body exercise (<10 min)

This exercise builds on the cognitive visualization and manipulation activities introduced in the *Astronaut Exercise* by extending the process to ostensibly anatomical images. Within an embedded video, a sequence of digitally produced still images of a transparent human body appearing to float in space are shown individually and then together in different orientations, suggesting rotation of the body through 360 degrees (Figure 3). Immediately following viewing of the images, learners are encouraged to close their eyes, imagine their own body or the transparent body moving in space, and to take conscious control of their visualizations to determine how their body, or the transparent body, moves in space.

##### Fork exercise (<20 min)

The first three exercises in *Exploring 3D Anatomy* can be considered together as training designed to support fundamental cognitive and physical spatial awareness. In the *Fork Exercise*, spatial activities in the *Astronaut Exercise* and *3D Body Exercise* are extended beyond mental visualization through the introduction of haptic and visual observation and spatial manipulation of a physical three‐dimensional object. The addition of a physical element within this exercise is intended to build upon the cognitive activities in the previous two exercises to strengthen learner three‐dimensional spatial awareness. In this exercise, the learner is invited to hold and rotate a fork (or similarly asymmetrically polarized object) through several orientations, while explicitly visually observing the fork as they move it around through space (Figure 3). If necessary, the fork can be substituted with any household object of a similar structure, including utensils, small tools, pens, or pencils.

##### Jar exercise (<55 min)

The *Jar Exercise* module is comprised of three tasks, the *Jar Exercise Introduction* (<15 min)*; Drawing on the Jar* (<10 min); and *Drawing the Jar onto Paper* (<30 min). Learners are given the flexibility to carry out the whole module within a single sitting, or to complete each section separately. Having developed basic observational and cognitive elements of spatial awareness within the first three exercises of *Exploring 3D Anatomy* for application to three‐dimensional anatomical structures, the *Jar Exercise* builds on these foundational skills. This exercise aims to support learners in their visualization of two‐dimensional cross‐sections of the axial, coronal, and sagittal anatomical planes, and application of their understanding of the planes to the three‐dimensional human body. This is achieved using two distinctly colored permanent markers to draw the coronal and sagittal planes onto the external surface of an empty glass jar (Figures 1, 4). Water colored with food dye (Figures 1, 4) is poured into the jar to represent the axial plane, with cooled weak tea (without milk) or other colored transparent beverages being suitable alternatives. Finally, the learner observes the jar using sight and touch (for example by tracing their finger over the marked lines while viewing and grasping the jar) and then draws the planes onto paper using differently colored pencils to distinguish each plane.

**FIGURE 4:**
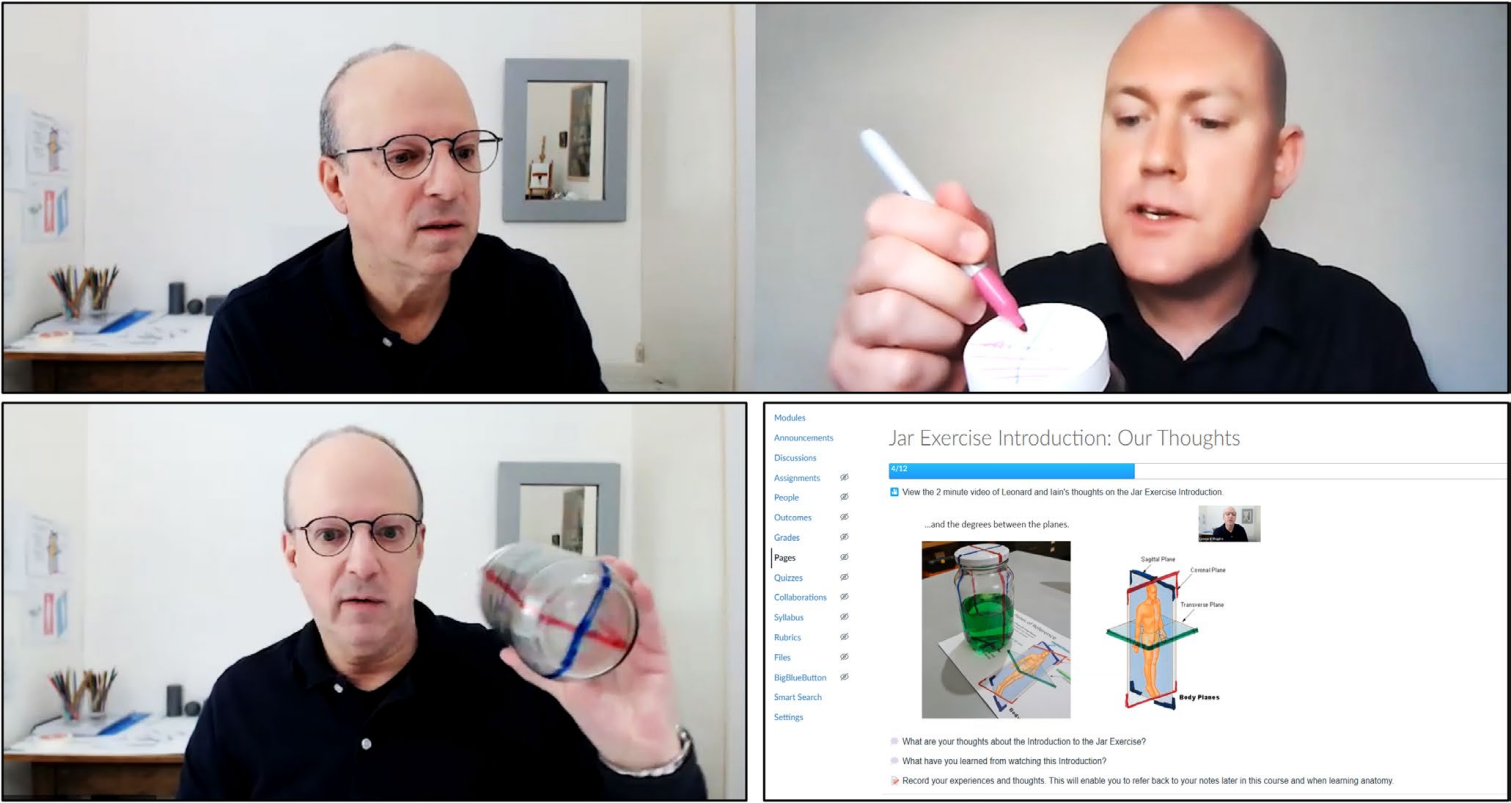
Screenshot images taken from embedded video demonstrations of the *Jar Exercise*, showing L.S. guiding I.K. in how to draw onto the jar to represent the coronal and sagittal planes (top), L.S. demonstrating completion of the ‘Drawing on Jar’ activity (bottom left). Screenshot from *Exploring 3D Anatomy* Canvas platform including a still image of a drawn‐on jar filled with colored liquid to represent the axial plane, a diagram to demonstrate the anatomical planes, and text‐based prompts for learner reflections on the exercise (bottom right).

##### Fruit exercise (>60 min)

The *Fruit Exercise* builds on knowledge and experience gained from the *Jar Exercise* to consolidate and expand learner understanding of the relationship between three‐dimensional anatomical structures and their representation as two‐dimensional anatomical planes. This exercise is underpinned by previous work of the authors, which describes the reciprocal cognitive two‐dimensional to three‐dimensional transition made by learners when interpreting anatomy in cross‐sectional images.^28^ In the *Fruit Exercise*, the body planes are drawn onto a citrus fruit, an object with an internal segmented structure, and which can be easily dissected. The citrus fruit is used to represent a three‐dimensional body or organ, so that when cut, the segments within the fruit appear in distinct arrangements depending on the plane in which the cut is made (Figure 5). This represents, for example, computed tomography images of the heart, where the level and plane of the scan affect the apparent size, shape, position, and number of the heart chambers and vessels that can be observed in the image (Figure 5). This analogy is emphasized by the educators within video demonstrations to support learners in applying the results of this activity to their understanding of anatomy. Learners follow the demonstration video, use permanent markers to draw body planes onto the surface of the fruit, use a knife to cut into the fruit along the planes, and observe the features of the dissected fruit using sight, touch, and drawing.

**FIGURE 5:**
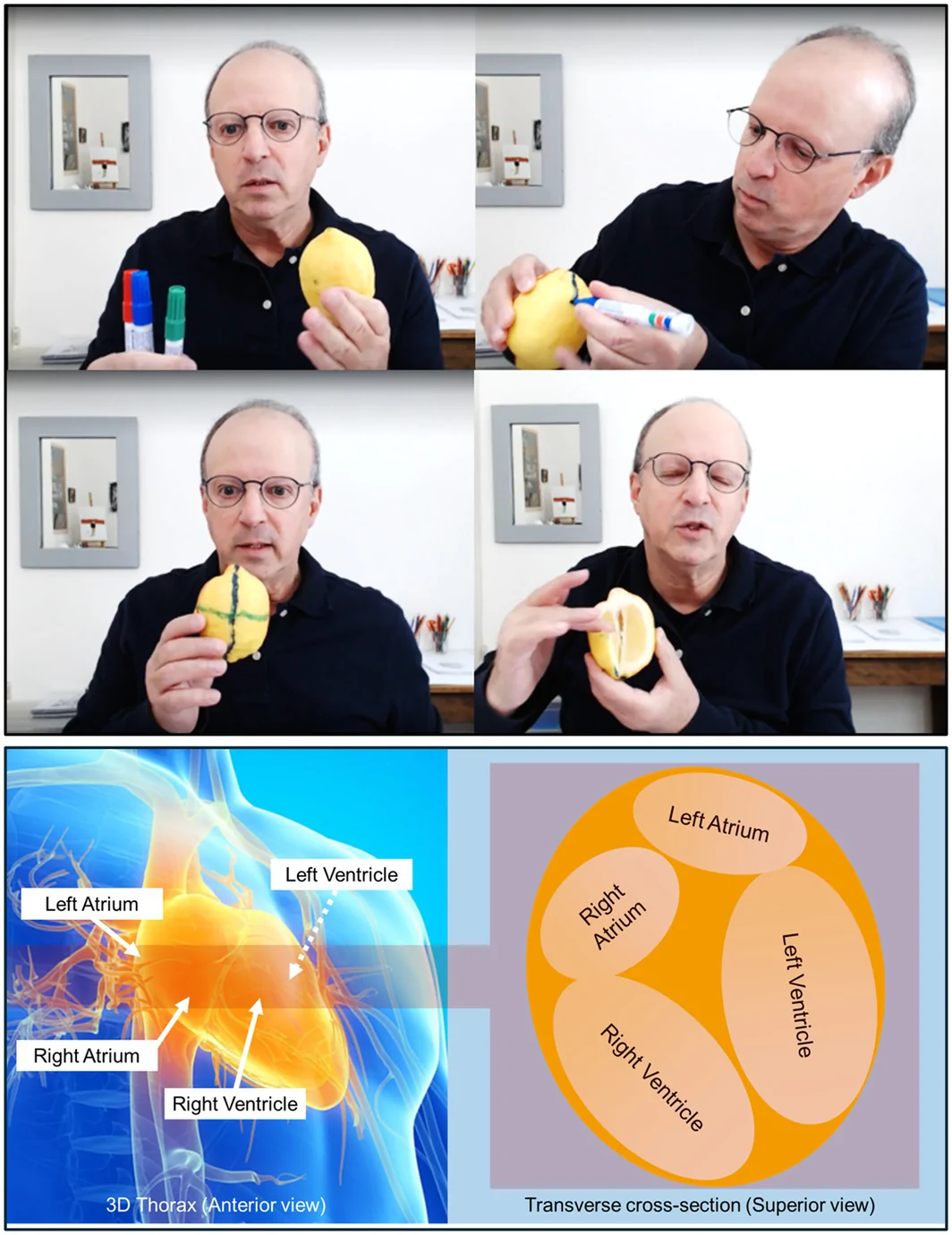
Screenshots from an embedded *Exploring 3D Anatomy* video, showing L.S. demonstrating how to carry out the fruit exercise using a lemon (top panel). Screenshot from *Exploring 3D Anatomy* demonstrating a labeled image of a three‐dimensional heart (image usage licence purchased from 123RF), analogous to a whole citrus fruit (bottom left) and corresponding transverse cross‐section through the heart chambers, analogous to the appearance of segments in a cut citrus fruit (bottom right).

##### Visual and haptic observation exercises (<10 min)

The *Visual Observation Exercise*, *Haptic Observation Exercise*, and the *Visual and Haptic Observation Exercise* are structured as separate modules within *Exploring 3D Anatomy* (Figure 1) but are described here together due to their collective purpose. These exercises extend beyond the implicit use of sight and touch in the previous exercises by encouraging learners to explicitly and purposively engage in visual and haptic observation activities, so they may gain multimodal sensory information from the objects being investigated.^12^, ^13^ Visual observation activities enable learners to identify specific spatial information, including the size, shape, position, and color of an object, while the addition of touch to their activities enables further sensory information to be gained, relating to the three‐dimensional form, texture, and weight of the object. In the *Visual Observation Exercise*, learners are directed to observe all physical aspects of an object (for example, a mug, cup, or glass, Figure 6) using sight only, for approximately 1 min. Learners can move their eyes, head, and entire body around the object in multiple directions in order to gain visual information from different perspectives.

**FIGURE 6:**
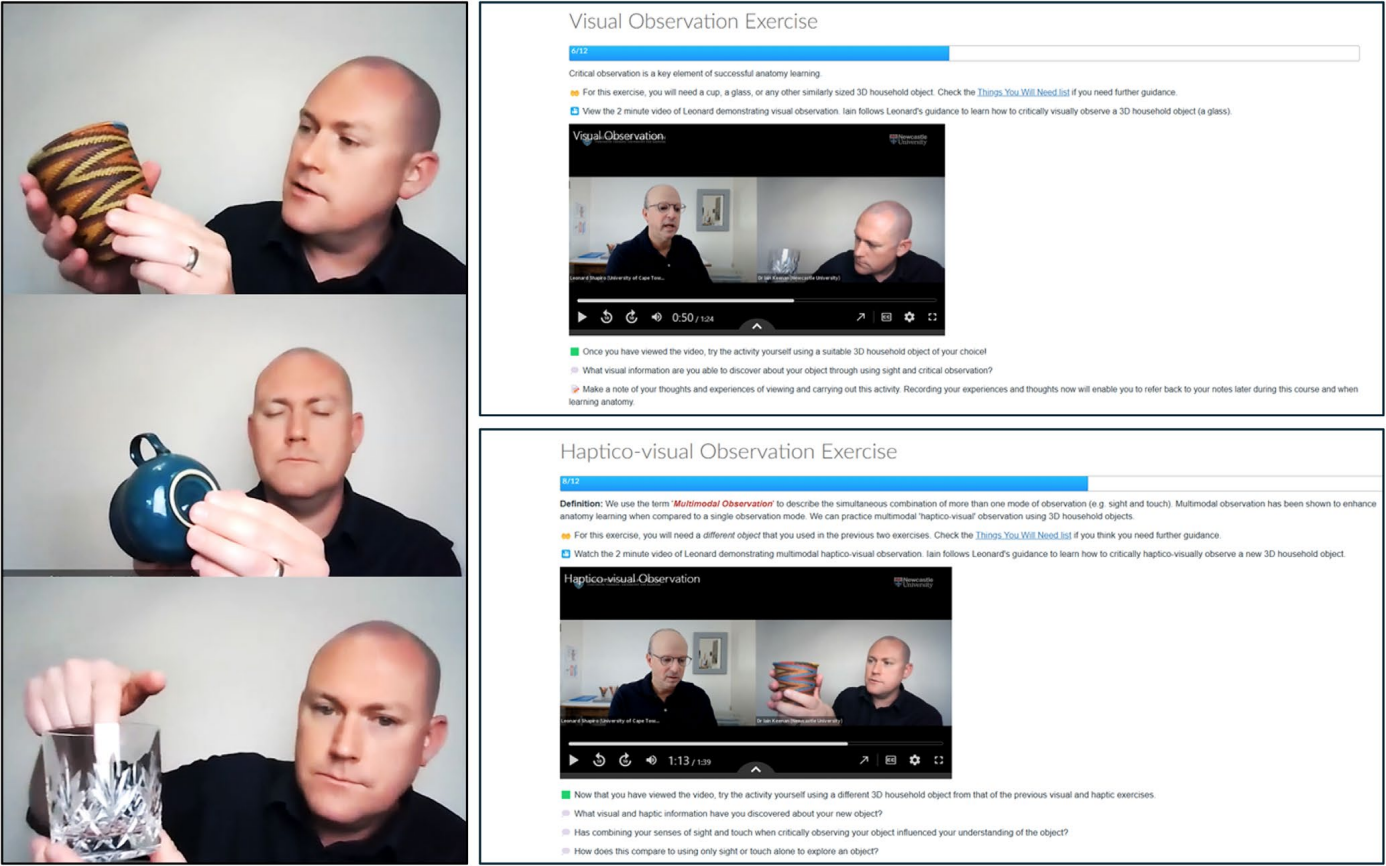
Screenshots of an *Exploring 3D Anatomy* embedded video (left panel), showing I.K. demonstrating haptic and visual observation of three similar but distinct three‐dimensional household objects (top to bottom). Screenshots from the *Exploring 3D Anatomy* course platform (right panel) show blue course progression bars, text‐based learner guidance and embedded videos of L.S. and I.K. demonstrating visual observation (top) and haptico‐visual observation (bottom).

The subsequent *Haptic Observation Exercise* requires learners to observe a similar but different object (for example a differently shaped mug, cup, or glass, Figure 6) using touch only. With their eyes closed, learners hold the object in their hands and actively apply exploratory procedures^13^, ^69^, ^70^ with their fingers by exploring the surfaces and spaces and by enclosing the object in their hand. The final exercise in this group builds on the previous two by combining sight and touch in the *Visual and Haptic Observation Exercise*. Learners now investigate another similar but differently shaped object (for example a third mug, cup, or glass, Figure 6) using both sight and touch. Learners are encouraged to consider and reflect upon each exercise, activity, and sensory modality in turn, both when used separately and when combined.

##### Haptico‐visual observation and drawing (>120 min)

The *Paper and Scribble Exercise* (<60 min) and the *Haptic and Visual Observation with Drawing Exercise (>60 min)* are presented here together as crucial elements of the Haptico‐visual observation and drawing method, as described in detail by the authors in their previous work.^12^, ^13^ These exercises build on the multimodal observational approaches that learners have practiced in the previous exercises, but now with simultaneous drawing of the object under haptic and visual observation. The *Paper Exercise* (manipulating sheets of paper in multiple different ways, for example, folding, tearing, crumpling, twisting) and the *Scribble Exercise* (making a variety of gestural marks on paper with a graphite stick) have been designed to support learners in increasing their confidence in performing directed, purposive, spontaneous, fluid, and varied upper limb movements. These actions that are required to successfully employ the Haptico‐visual observation and drawing method.^12^, ^13^ The ability of learners to effectively engage in the *Haptic and Visual Observation with Drawing Exercise* is the culmination of the *Visual and Haptic Observation Exercises*, and the *Paper and Scribble Exercise*. Learners use one of their hands to hold and feel a three‐dimensional object (e.g., a ballpen hammer, Figure 2), while with the other hand the learner simultaneously makes marks (draws) on paper that reflect the sensory information gained from the exploratory procedures of the observing hand.^12^, ^13^

##### Box exercise (<25 min)

The final exercise of *Exploring 3D Anatomy* enables learners to bring together and apply their spatial, visual, and haptic skills and understanding gained from the previous course exercises. The *Box Exercise* (Figure 7) is a haptic observation exercise combined with visual elements, which focuses on an experiential comprehension of three‐dimensional space via direct physical practice. To achieve this, the outside (Figure 6A) and the inner (Figure 7) surfaces and spaces of an empty box (for example, a shoe box) are explored using one hand. The learner may intermittently open their eyes during the process in order to visually observe the movement of their hand, but their eyes should primarily remain closed to reinforce haptic perception as their primary sense modality. Following initial haptic exploration and observation, and with the eyes opened, the learner uses their non‐exploring hand to simultaneously make graphite marks on paper, which reflect the multiple trajectories made by the exploring hand (Figure 7). This exercise is designed to develop and strengthen learner spatial understanding with respect to the internal and external volumes of anatomical structures, for example, the abdominal and thoracic cavities, or the skull.

**FIGURE 7:**
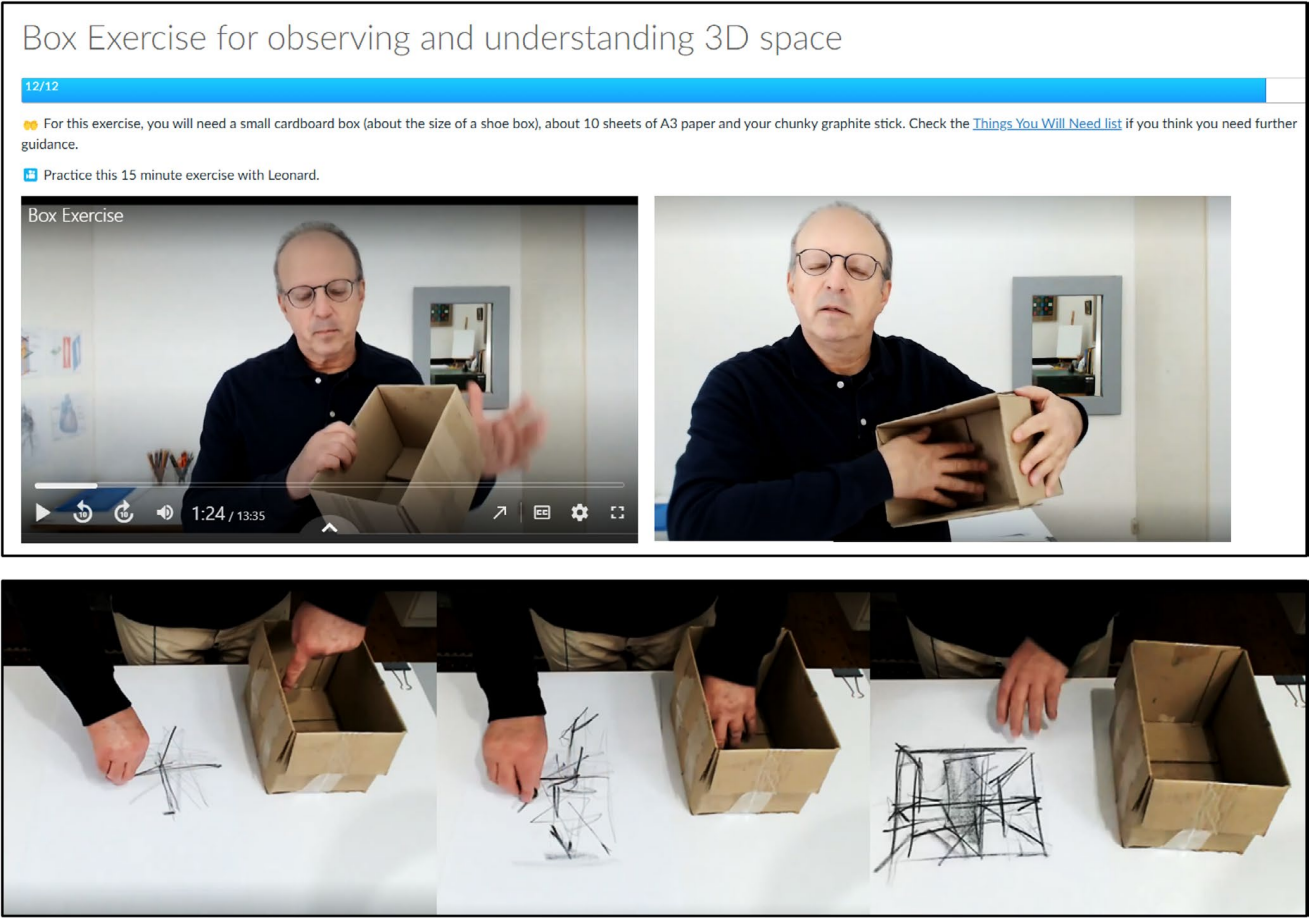
Screenshot of *Exploring 3D Anatomy* showing course progress bar (blue), text‐based guidance for learners, and an embedded video showing L.S demonstrating the *Box Exercise* (top panel). Screenshots of embedded *Box Exercise* video demonstrations showing L.S. demonstrating, visual and haptic observation of the box and progressive (bottom, left to right) haptico‐visual observation and drawing of the box.

### Phenomenological investigation of student experiences of exploring 3D anatomy

A convenience sampling approach was implemented to recruit student participants from different years of study on the M.B.B.S. program at Newcastle University, and who were available, known to, and contactable by, the authors during the 2024 summer vacation period. Focus Group 1 (*n* = 6) was held in July 2024 with medical student participants, during a period of low M.B.B.S. student activity on *Exploring 3D Anatomy* discussion boards. Following data collection, participants were assigned anonymous identifiers as follows: Participant A (Year 4, Graduate‐Entry); Participant B (Year 4, Undergraduate); Participant C (Year 4, Undergraduate); Participant D (Year 2, Undergraduate); Participant E (Year 4, Undergraduate); Participant F (Year 2, Undergraduate). Focus Group 2 (*n* = 2) was held in November 2024 to ensure comparable representation from preclinical Year 1 and 2 students, and to capture views during a period when M.B.B.S. student activity on *Exploring 3D Anatomy* course discussion boards was greater. Following data collection, participants were assigned anonymous identifiers as follows: Participant G: (Year 1 Undergraduate); Participant H: (Year 2 Undergraduate). Focus group methods and sample sizes aligned with previous work by the authors^12^, ^13^, ^14^ and were based on established focus group principles.^71^, ^72^, ^73^

### Data analysis

Combined interpretative phenomenological analysis^74^, ^75^ was implemented to construct themes based on the personal lived experiences of participants, in order to explore the richness and depth of their perceptions around anatomy education and *Exploring 3D Anatomy*. Preliminary coding was performed by authors J.Z. (Focus Group 1) and C.M. (Focus Group 2). Double‐coding of both focus groups was then performed independently by authors I.K. and L.S. Codes constructed from each focus group transcript were combined and collaboratively constructed into themes by I.K. and L.S. The authors acknowledge, as the creators and educators of *Exploring 3D Anatomy*, that they are invested in the pedagogic value of the course, and that their judgments and biases are present in the analysis and interpretation of the data, which are essential elements within the analysis approach used.^74^, ^75^

### Ethical assessment

The Newcastle University Faculty of Medical Sciences ethics committee provided institutional ethical approval (2289/18929/2022) and this work was approved by the Newcastle University School of Medicine Research Management Group for undergraduate medical student participation in research. All participants provided their informed consent to participate, were advised regarding the purpose of the research, and were made aware that they were free to withdraw at any time. Information and assurances with respect to confidentiality and security of their data were provided to participants, who were assigned identification codes (Participant A–H) prior to analysis to ensure individual participants could not be identified by researchers. To incentivize participation, Newcastle University School of Medicine certificates were awarded to recognize their contributions.

## RESULTS

From interpretative phenomenological thematic of focus group transcripts, a nested set of four themes was constructed (Figure 8). Based on discussions during focus groups, the first, overarching theme represents contrasting views of participants with respect to their approach to learning. Each subsequent theme then represents distinct subsets of participant perceptions that fall within the previous theme. Themes can be superficially described as characterizing dichotomous viewpoints that are in direct conflict. However, on closer inspection, a more nuanced spectrum of views between each extreme may be identified.

**FIGURE 8:**
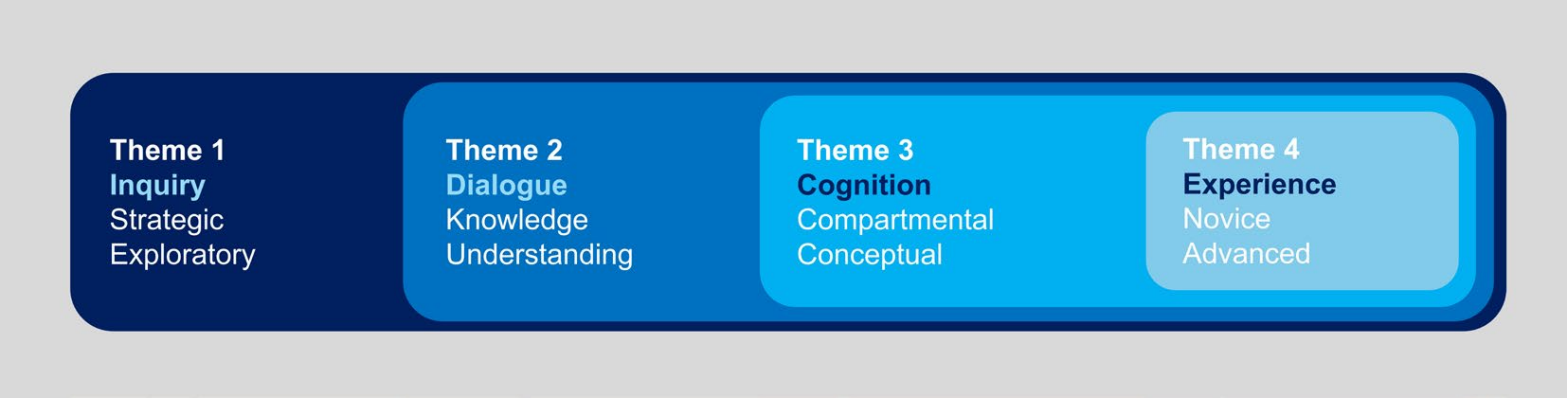
Nested set of four themes (Inquiry; Dialogue; Cognition; Experience) constructed from combined interpretative phenomenological analysis of two focus group transcripts. Each respective theme was constructed as a dichotomy or spectrum between extreme viewpoints (Strategic–Exploratory; Knowledge–Understanding; Compartmental–Conceptual; Novice–Advanced).

Disagreements occurred during Focus Group 1, while participants in Focus Group 2 were largely in agreement. The authors found that disagreements among participants provided a deep and rich understanding of the issues and welcomed and valued comments from these opposing viewpoints. While specific views and comments have been grouped into themes and sub‐themes, individual participants have not been categorized as holding a singular view. This approach allows for nuance, ambivalence, and fluctuation in the perspectives of individuals. Some comments made by the same individual have been combined to construct example comments below.

1. Inquiry: Strategic–exploratory

This theme was constructed from author interpretation of participant comments regarding their approaches to anatomy learning and their views of *Exploring 3D Anatomy*. “*Strategic inquiry”* focused on anatomy knowledge in absolute and objective terms and described learning approaches that participants considered would directly improve their assessment performance and progression. In contrast, “*exploratory inquiry”* demonstrated participant metacognition, and the value placed in discovery and subjective interpretation of anatomy as a route to holistic understanding of anatomical concepts. Tentative *Inquiry* stances are described as “*Exploratory‐Pragmatic”* when anatomical conceptualization was valued by participants but was perceived as being constrained by what was realistically achievable within their own circumstances.

1.1 Exploratory: *“I think the thing about anatomy is, I think we often see it as a rote learning exercise, when I think it should be something that you can work out. Just because you're not discovering something new doesn't mean that you're not working it out and seeing things that are maybe a bit different. You're just working out what's happening, based on what you're seeing”*. [Participant C, Year 4 Undergraduate, Focus Group 1]

1.2 Exploratory‐pragmatic: *I would have loved to have the chance to think about conceptualizing things a little bit more. I feel like if at* [the start of year 1] *I would have been like, “well I can't learn everything, what is the most sacrificable part of the course?,” and it probably would have been the conceptual things. And that's probably why I didn't really engage with* [*Exploring 3D Anatomy*] *at* [the start of year 1].’ “[*Exploring 3D Anatomy*] *is not an activity that's aiming to teach people in a binary way. It's aiming to broaden people's ways of learning, so it's not going to work for everybody”*. [Participant A, Year 4 Graduate‐Entry, Focus Group 1]

1.3 Exploratory‐pragmatic: *Some of the more abstract concepts, like the* [*Astronaut*] *exercise, I know encourages you to think about how you can apply this to your learning. But I think, especially with medical students, there's this idea that if this isn't going to be helping me, I'm not going to do it.’* [Participant D, Year 2 Undergraduate, Focus Group 1]

1.4 Strategic: *‘For me, a lot of the* [pre‐clinical M.B.B.S.] *anatomy was just about trying to remember labels to pass exams, or what a nerve does, not necessarily understanding the anatomy, and where it actually is. When you've not got much time, you do want to be doing things that ultimately are going to help you pass your exams.” “‘It's not as much about how long [an online resource] is for me, it's about how relevant the content is”*. [Participant B, Year 4 Undergraduate, Focus Group 1]

2. Dialogue: Knowledge–understanding

This theme focuses on disparate views of anatomical education in terms of the focus and value placed on *dialogue* around the memorization of facts (*knowledge*) versus holistic and conceptual comprehension (*understanding*) in the context of learner reflection and discussion. How “reflection” is defined is also considered here, as the term can be understood and interpreted by participants in the context of their medical training, and in a broader sense, where it describes contemplation of prior experiences. Construction of Theme 2 includes elements encompassed by Theme 1 inquiry stances. From a s*trategi*c *inquiry* perspective, anatomy is viewed as an objective, factual discipline, where discussion of anatomy is focused on “*knowledge”* gains, and learning and assessment of anatomy are therefore outside the scope of reflection and interpretation. In contrast, “*exploratory inquiry”* considers dialogue around these subjective elements as important for facilitating appreciation of anatomical concepts, which in turn leads to holistic “*understanding”* of anatomy.

2.1 Understanding: *Discussion [is valuable] for drawing or feeling the household object. You're feeling, you're grasping these household objects, and you're trying to get something out of it. You're trying to learn more about how to understand an object. I feel in a situation like that, it is more useful to say what you got out of it, like “Oh, this really helps me understand specific contours of this object, I had better abilities when visualizing it afterwards,” rather than something with the planes where it's like “oh, I understand the planes now.”* [Participant D, Year 2 Undergraduate, Focus Group 1]

2.2 Understanding: *“They [discussion boards] might not have new information when it comes to the science, [but] they're a really good place to learn mnemonics and acronyms and stuff, it's a good way to share ways to learn, if not the actual learning itself.”* [Participant E, Year 4, Undergraduate, Focus Group 1]

2.3 Understanding: *“I do see the value of…reflecting and discussing the things that people said* [in discussion boards], *which then helps me to understand a bit more or help me to think about the benefits of the* [*Exploring 3D Anatomy*] *exercises. Maybe like if there's more of an emphasis on ‘discussion’ rather than ‘reflection.’ ‘Reflection’ is a bit of a loaded concept [in medical school]. So maybe more of an emphasis on discussion and learning from each other rather than personal reflection [would be useful]”*. [Participant D, Year 2 Undergraduate, Focus Group 1]

2.4 Understanding: *“There is a lot of reflection in* [*Exploring 3D Anatomy*], *and at the start of the [M.B.B.S. degree curriculum], you get overwhelmed with the amount of reflection you've got to do. So, if I were to open up a course* [like *Exploring 3D Anatomy*] *and see ‘And now reflect again,’ that would definitely put me off. The people who are really valuing the experience [of Exploring 3D Anatomy] will be reflecting on it anyway.”* [Participant A, Year 4 Graduate‐Entry, Focus Group 1]

2.5 Knowledge: *“You don't want someone to have an experience [of Exploring 3D Anatomy] and get a particular point out of it, and then someone else to say something completely different [on the discussion board], and the first person to feel like they didn't learn the right thing. When you get to clinical years, everybody's using practice question banks and going on discussion boards to discuss the answers and give hints and tips of how people reached answers, rather than to discuss reflected learning points”*. [Participant A, Year 4 Graduate‐Entry, Focus Group 1]

2.6 Knowledge: *“I don't think [discussion boards are useful], because for anatomy it's like it's either right or isn't, because most anatomy has already been discovered*…*it's not like I'm going to learn from what someone else imagined. But [with] the planes on a 3D object, I think discussion there could be helpful, because people might not understand certain planes. If there was a larger group of people using [Exploring 3D Anatomy discussion boards], then it makes sense to actually use it as well, because then you'd actually see responses”*. [Participant F, Year 2 Undergraduate, Focus Group 1]

3. Cognition: Compartmental–conceptual

This theme was constructed from comments regarding learner *cognition*, that is, how participants think about anatomy learning in a broad sense, in addition to their specific three‐dimensional spatial conceptualization through visual and haptic observation in *Exploring 3D Anatomy*. *Compartmental* refers to participant allocation of learning into particular areas that they feel are directly relevant to them, particularly the explicit application of anatomical learning to taught content and clinical placements delivered within the context of the formal medical curriculum. This process of compartmentalization strongly aligns within the *strategic* and *knowledge* extremes of Themes 1 and 2, respectively. In contrast, *conceptual* here refers to a focus on holistically observing and assimilating ideas, as a distinct *exploratory* route that seeks to achieve *understanding* of three‐dimensional space for application to anatomical size, shape, positions, and relationships. Based on this interpretation, Theme 3 is assigned as a nested subset of Themes 1 and 2.

3.1 Conceptual: *“One thing I struggle with is nerves and veins and arteries, because it's harder to visualize their position, especially with [anatomical] variations. I found it harder to learn where things went and what they did, just because of that variation. I found it a lot easier to learn things like muscles, because they're bigger structures, which were easier to get my head around”*. [Participant D, Year 2 Undergraduate, Focus Group 1]

3.2 Conceptual: *When you're drawing it out, it does kind of help you visualize it a bit better because it's a bit more of an active method of learning; active learning is better than passive. So that drawing it out method does really help. Finding the different sinuses behind the heart, maybe [Exploring 3D Anatomy] could be useful, or at least the visualizing techniques of [Exploring 3D Anatomy]. For some parts of anatomy that were a bit harder to understand in 3D space, [Exploring 3D Anatomy] helped [me] figure out new methods to do that: the coronary arteries and veins, and lower gastrointestinal because there's so many structures in that small space*. [Participant H, Year 2 Undergraduate, Focus Group 2]

3.3 Conceptual: *Some of the drawing and haptic visual observation [in Exploring 3D Anatomy] did help my observation, being able* to *pick out details I wouldn't have noticed just by looking at something and applying that to how I learned anatomy. There were times [in the anatomy laboratory] where I was just looking at [donor specimens], and I didn't particularly want to touch the specimens. But doing [Exploring 3D Anatomy] made me realize there were so many details I missed, just by observing the objects, which [were details] I could then pick up on by feeling. I could get* a *better sense of proportion and scale when I added in that extra touch*. [Participant D, Year 2 Undergraduate, Focus Group 1]

3.4 Conceptual: *The Astronaut [Exercise] I found particularly interesting; I was imagining [the nervous system] while I was spinning around and trying to picture every single part of it, which I think is really useful because it's not just seeing the frontal view or the posterior view; you're seeing the whole thing rotating around. Throughout [Exploring 3D Anatomy], you've had to do drawings; you have to draw a lot of different things. Better skills of drawing, even if they're very bad basic diagrams, could help aid my understanding. Imagining yourself feeling as you draw it at the same time, the concentration you have, you definitely remember the structures a lot better, like the ridges along certain structures and like texture. It's good to just piece it all together*. [Participant G, Year 1 Undergraduate, Focus Group 2]

3.5 Compartmental: *“The [Astronaut Exercise] was, ‘imagine yourself in space,’ which is good for what it's trying to do, but there's no real relating it into anatomy, or relating it into the medical school curriculum, or at least they don't explain how it can help you”* [Participant B, Year 4 Undergraduate, Focus Group 1].

3.6 Compartmental: *“I feel [Exploring 3D Anatomy] needs to be contextualized a little bit more to look at anatomy. I know there was the bone and things like that, but there was too much focus on household objects. When you're looking at the fork activity, then [Exploring 3D Anatomy could say] ‘let's look at an organ in that way.” In some ways it's spelling it out a little bit too much, but it does help with the actual anatomy learning as well as just the method of learning*. [Participant A, Year 4, Graduate‐Entry, Focus Group 1]

4: Experience: Novice–advanced

This theme has been constructed as a nested subset within Themes 1, 2, and 3. Participants provided their views of the relative value of *Exploring 3D Anatomy* to distinct target audiences, describing how they felt *Exploring 3D Anatomy* would be most valuable for *novice* students, who are in the early stages of their anatomy studies. There was a consensus that new learners would benefit from the *exploratory conceptual understanding* provided by *Exploring 3D Anatomy* in its current form. In contrast, this theme also shows how *advanced* Year 4 participants, currently in their clinical years of study, felt that *Exploring 3D Anatomy* would require modifications to focus on the *strategic* delivery of *compartmentalized knowledge*. Participants characterized the latter view as the application of anatomy to real‐world clinical and surgical scenarios.

4.1 Novice: *“I definitely think [Exploring 3D Anatomy] highlighted stuff which helps with learning, and especially with the haptic learning that was really interesting. [Exploring 3D Anatomy] would have been helpful to have [been] done earlier on [in their M.B.B.S. program curriculum]*, *especially stuff that we take for granted…like the different planes; coronal plane, sagittal plane, transverse plane, where [Exploring 3D Anatomy] did provide quite a helpful introduction to those concepts.”* [Participant E, Year 4 Undergraduate, Focus Group 1]

4.2 Novice: *“It feels like if you're going to do* [*Exploring 3D Anatomy*] *as a medical student, it needs to be very early on when you're first learning basic anatomical terms and about the planes*. *It would be useful if you had [Exploring 3D Anatomy] as an induction‐type activity. When you're first starting with anatomy and having it as a group activity where you try these things* [*Exploring 3D Anatomy* exercises], *and then you discuss them and reflect on them, and how you could use them, it would feel more beneficial than a solo activity”* [Participant A, Year 4 Graduate‐Entry, Focus Group 1]

4.3 Novice: *I did find* [*Exploring 3D Anatomy*] *useful, and something which I did wish I'd done* [at the start of Year 1], *but for that length [of course] it almost feels like something which you'd want to do before you went into medical school’*. [Participant D, Year 2 Undergraduate, Focus Group 1]

4.4 Novice: *If this is a first‐year student at the very start of the [M.B.B.S] degree* [the emphasis placed on reflection in *Exploring 3D Anatomy*] *might be quite daunting for them, and they might end up paying more attention to their reflections than they are to the learning’*. [Participant E, Year 4 Undergraduate, Focus Group 1]

4.5 Advanced: “*I feel* [*Exploring 3D Anatomy*] *needs to have more clinical application, if people* [are to see] *it as valuable. If you made changes, I think it would be more clinical contextualizing. The big* [anatomy‐related] *thing we are exposed to in clinical years is laparoscopic surgery, so a video running through anatomy from the laparoscopic perspective…would be really helpful”*. [Participant A, Year 4 Graduate‐Entry, Focus Group 1]

## DISCUSSION

*Exploring 3D Anatomy* is a free supplementary online course provided to medical students at Newcastle University to coincide with their first experiences of formal anatomy education during their M.B.B.S. program curriculum, with the aim of supporting their spatial awareness so they may apply this understanding to an appreciation of three‐dimensional anatomy. Findings from this phenomenological study have enabled the construction of four nested themes, which highlight differences in the philosophical positions of learners with respect to their own study of anatomy and that of their peers: the importance and meaning behind the processes of internal reflection and external discursive interactions, the strategic compartmentalization and holistic appreciation of anatomical concepts, and universal support for the provision of *Exploring 3D Anatomy* to novice learners.

The themes constructed from this investigation of *Exploring 3D Anatomy* have served to highlight wider tensions present between varying levels of student intrinsic motivation to learn and conceptualize anatomy, and the extrinsic burdens that are placed on them when studying anatomy at medical school. Findings here are supported by the concept of students as ‘deep,’ ‘surface,’ and ‘strategic’ learners who are motivated intrinsically, extrinsically, and by fear of failure, and by achievement respectively.^76^ Additionally, learning profiles (active, collaborative, structured, passive), which describe differing student motivations and learning strategies that vary between individuals, cohorts, curricula, and institutions in both medicine^77^ and anatomy,^78^ can inform interpretation of results. Nonetheless, the themes presented here offer further valuable insights beyond these ideas into the specific context of anatomy learning and *Exploring 3D Anatomy* from the medical student perspective.

### Learner inquiry: Strategic–exploratory

The first key area to consider concerns conflicts between the *strategic* and *exploratory* inquiry approaches of learners when studying anatomy within the context of a medical curriculum. Learner career goals when choosing to study medicine or other healthcare professions in pursuit of a clinical vocation, the reasons behind their motivation to study, and their self‐awareness and metacognition when studying anatomy all require scrutiny to fully interpret this theme.

Medical student motivations are likely to differ with respect to their non‐clinical biomedical and life sciences counterparts, not only because medical training is specifically targeted at a single professional vocation, but also because the discipline involves higher levels of workload and responsibility, which can lead to anxiety, stress, and burnout.^79^, ^80^ This distinctive quality of medicine is highlighted in comment 1.4, where medical students are viewed as ‘*especially’* focused on *strategic* forms of inquiry.

Medical students primarily choose their discipline for altruistic reasons, to help patients and support communities^81^, ^82^, ^83^ and may therefore request explicit links to be made between learning activities and their underlying motivations to optimize their emotional, behavioral, and cognitive engagement.^50^ Favorable medical student perceptions of anatomical education are heavily driven by the perceived clinical relevance of the subject^84^, ^85^, ^86^ and are likely to have not only explicitly (comments 1.4, 4.5) but also implicitly influenced participant perceptions of *Exploring 3D Anatomy*. Moreover, medical students assign value to the clinical relevance of assessments,^87^ a finding supported by self‐determination theory, which describes the importance of ‘relatedness’ for intrinsic motivation^88^ where a connection to clinical relevance may foster belonging within the medical student community.^89^

Well‐intentioned holistic learner philosophies seeking to explore anatomical concepts (comments 1.1, 1.2, 1.3) may be challenged by the perceived clinical relevance, volume, intensity, and complexity of medical content (comment 1.2), which includes their ability to manage work–life balance,^80^, ^90^ and crucially, their need to perform in high‐stakes summative assessments (comment 1.4).^87^ Due to these factors, some learners may *pragmatically* choose to sacrifice *exploratory* activities like *Exploring 3D Anatomy* in favor of *strategic* inquiry. However, while assessment, at least in part,^91^ may drive learning,^87^, ^92^ a *strategic* approach may not necessarily be most effective for those motivated by achievement. Self‐regulated learning theory and mastery goal orientation are characterized by *exploratory* attributes including openness and metacognition, and are key to lifelong learning,^93^ which is crucial for progression in medicine and health care professions.^94^, ^95^ Meanwhile, depending on the context, employing *exploratory* learning approaches that incorporate critical thinking and elaboration of understanding around anatomical relationships and clinical applications may^96^ or may not^97^ positively correlate with assessment performance.

An additional consideration not mentioned by participants here is the potential impact on mental health due to the multiple extrinsic pressures that are placed on medical students,^79^ and the potential for *Exploring 3D Anatomy* to add to this load. Although participants did not explicitly voice concerns in this area, it is hoped that the relatively low time commitment and flexible design of the course would not be detrimental in this respect. Furthermore, engaging in holistic extra‐curricular art‐based activities such as *Exploring 3D Anatomy* may actually serve to enhance their psychological well‐being, reduce stress, and nurture recovery within a supportive online learning community.^98^, ^99^, ^100^, ^101^

### Learner dialogue: Knowledge–understanding

Findings within this theme present a clear divide between perspectives that welcome discussions around uncertainty and exploration in anatomy learning, and those views that either do not embrace value in such dialogue or only do so from an assessment‐driven *strategic* viewpoint. Additionally, misinterpretation of the definition and nature of “reflection” in the context of *Exploring 3D Anatomy* has yielded valuable insights into how students view undergraduate medical and anatomical education and their approaches to it.

With respect to *Exploring 3D Anatomy* discussion boards, participant comments 2.5 and 2.6 under this theme highlight a wider issue of *knowledge*‐driven assessments in medical education, in which students may be implicitly encouraged to ignore the uncertainties and ambiguities inherent in medical practice.^102^, ^103^, ^104^ As a result, students are likely to assume that any medical question must have an absolute, single correct answer. This idea extends to the anatomical sciences, where although standard models of the human body are typically presented in textbooks and digital applications, there are a vast number of variations from these norms in the anatomy and pathology of individual patients, as noted here (3.1). *Exploring 3D Anatomy* has been designed with this uncertainty in mind, to support learners in their personal interpretation of visualization and observation tasks. In this way, the course is the antithesis of the idea that anatomy is “*either right or isn't”* (2.6), as it does not expect students to learn only “*the right thing”* (2.5). Rather, *Exploring 3D Anatomy* encourages learners to “*share ways to learn”* (2.2), discuss “*what someone else imagined”* (2.6), and to help learners “*to understand a bit more”* (2.3), to foster collaborative reflection and metacognition in the construction of new perspectives such as the “*the planes on a 3D object’* (2.6), the “*specific contours of this object”* and also “*better abilities when visualizing it”* (2.1). Nonetheless, it is clear from the *knowledge* responses in this theme, that learners with *strategic* perspectives may not appreciate, value, or feel they have the capacity to approach anatomy learning dialogues with the aim of achieving *understanding*.

This theme has highlighted further areas of development of discussion boards within *Exploring 3D Anatomy*, where a more deliberate instructor presence and leadership, explicit dialogue‐based tasks and learning goals, and the setting of boundaries and conditions, while minimizing barriers for learner contributions^51^ can provide an enhanced experience for learner participation. Amendments and clarifications around the use of the term “reflection” are also required. It is clear from participant comments that the process of “reflection” is widely viewed *strategically* as a course requirement and can result in a specific form of fatigue,^105^ rather than forming the intended *exploratory* opportunity for metacognition and understanding.^106^ Based on this finding, the creators have now deliberately removed the term “reflection” from the exercises and activities within *Exploring 3D Anatomy* and instead ask learners to “share their thoughts and experiences.” Embedded educator “Reflection” videos have also been retitled as “Our Thoughts.” It is hoped that this simple change in terminology will separate *Exploring 3D Anatomy* from mandatory curriculum requirements and therefore foster a more *exploratory* approach among learners.

### Learner cognition: Compartmental–conceptual

This theme further emphasizes the disparity between learner perspectives relating to how they think about learning and their views on the approaches and purpose of studying anatomy, now specifically relating to ideas around three‐dimensional spatial awareness, understanding, and skills in anatomy and, in turn, medical practice. Of particular note were the contrasting comments (3.4 and 3.5) surrounding the Astronaut Exercise, a task which was perceived as valuable from both a *conceptual* and *compartmental* perspective, but was criticized within the latter stance, from a *strategic*‐*knowledge* point of view, for lacking direct relevance to a curriculum‐driven *compartment*.

Activities for encouraging development of metacognitive skills in anatomy learning have been described in previous work, including the use of reflective discussion,^107^ reflective writing,^108^ and art‐based techniques,^21^, ^109^ all elements provided within *Exploring 3D Anatomy*. Consequently, it could be anticipated that learners who began with a *compartmental* stance may move closer toward a *conceptual* perspective as they progress through the course. Indeed, *Exploring 3D Anatomy* has supported metacognition by helping one participant to “*figure out new methods”* of conceptualizing three‐dimensional spatial anatomy (3.2), another to appreciate the value of haptico‐visual observation and drawing course activities for “*being able pick out details I wouldn't have noticed,”* in realizing there were “*so many details I missed”* and “*applying that to how I learned anatomy”* (3.3), and another to recognize value in visualizing the human body as a “*whole thing rotating”* in space, drawing as a cognitive exercise, and the focus and “*concentration you have,”* which helped them “*definitely remember the structures”* and “*piece it all together”* (3.5).

Nonetheless, it may be necessary to provide a more structured approach to metacognitive training^110^ around spatial concepts, in addition to making the links to anatomy and the intended benefits of the course more explicit. Doing so may encourage learners with “*compartmental”* perceptions, who feel that there is “*no real relating it into anatomy, or…the curriculum”* (3.5) or that the course requires further contextualization “*to look at anatomy”* with a reduced “*focus on household objects”* (3.6) to make the conceptual and metacognitive leap required to fully benefit from *Exploring 3D Anatomy*. Such learners, who do not immediately appreciate the value of, for example, the Astronaut Exercise, or the Fork Exercise, may benefit from performing these tasks with anatomical images or models in the laboratory. These learners will then be able to take personalized ownership of their learning, by applying broad *conceptual* principles of spatial awareness and spatial understanding to their own study of anatomy, which may in turn result in increased autonomy, competence, and motivation,^88^ and the enhanced assessment performance they desire.^111^

That anatomy learners possess differing levels of visual and haptic spatial abilities that can influence their assessment performance is an established idea in anatomical education.^17^, ^19^, ^22^, ^26^, ^35^, ^40^ For this reason, *Exploring 3D Anatomy* aims to enhance the spatial awareness of learners at all ability levels. Although not explicitly mentioned by participants here, it is possible that there is an implicit link between “*strategic‐compartmental”* learners and lower levels of spatial awareness, ability, understanding, or skills,^36^, ^112^, ^113^ an idea which may explain differences in learner *compartmental* and *conceptual* perspectives of *Exploring 3D Anatomy* and their wider reliance on, for example, three‐dimensional learning resources or practice assessment questions respectively. Meanwhile, it is important to note that aphantasia, the inability for individuals to visualize images in their “mind's eye”,^114^, ^115^, ^116^, ^117^ has been identified as a lack of sensory imagery, rather than a deficit in metacognition,^116^ and so is unlikely to influence focus on either a *conceptual* or *compartmental* perspective.

### Learner experience: Novice–advanced

Having shown that learners who engage with anatomy learning from an *exploratory‐understanding‐conceptualization* perspective find the most value in *Exploring 3D Anatomy*, participant views also suggest that greater benefits can be gained from the course by inexperienced *novice* learners and support the integration of *Exploring 3D Anatomy* at the point of induction to (4.1, 4.2, 4.4) or before the start of (4.3) formal medical training.

Learners particularly highlighted the value of the course for supporting *novice conceptual understanding* as “*an induction‐type activity”* when learning “*basic anatomical terms”* (4.2) *and* for introduction to “*the different planes”* (4.1). Novice learners are likely to have lower levels of spatial ability^23^ and competence in three‐dimensional visualization^118^ than more advanced learners, due to these skills being developed through exposure and education, and are therefore more likely to benefit from the spatial training provided by *Exploring 3D Anatomy*. Indeed, the authors have previously identified the value of introducing three‐dimensional and cross‐sectional conceptualization to new medical students,^26^ while cognitive function also develops with experience of drawing and art‐based practice.^119^, ^120^, ^121^

Participant comments under this theme also revisit potential drawbacks relating to reflective *dialogue* and *compartmental cognition* around clinical relevance, in terms of their respective importance for new and experienced learners. There is an agreement of both students and educators that anatomy knowledge is essential for clinical practice,^122^ but also a widespread idea that medical student anatomy knowledge is lacking and may impact patient safety,^123^ Here, a Year 4 participant with an *advanced* viewpoint (4.5) identified a need for *Exploring 3D Anatomy* to have *“more clinical application,”* specifically relating to videos of *“anatomy from the laparoscopic perspective.”* However, the inclusion of such higher level realism and dynamic anatomical representations is beyond the intended scope of *Exploring 3D Anatomy*, which is to support fundamental spatial understanding. Adding detailed surgical elements would likely be counterproductive, resulting in increases in both extraneous and intrinsic cognitive load,^17^, ^124^, ^125^ with negative impacts on preclinical students already grappling with anatomical conceptualization and the wider cognitive challenges experienced by novices.^126^ Furthermore, in the context of the proportion of medical students choosing surgical careers,^127^ focusing heavily on surgical anatomy from the outset is unlikely to be inclusive for all learners. Consequently, with an *advanced* target audience already in mind, the authors have created Exploring 3D Anatomy Plus, an extension of the original course that is specifically aimed at educators, clinicians, and health care professionals, which will also benefit medical students in their clinical years who are interested in pursuing surgical careers. Indeed, *Exploring 3D Anatomy Plus* includes a specific “Surgical Instrument Exercise” that targets important spatial skills when manipulating laparoscopic tools,^128^, ^129^ and there is scope to include further course activities that support interpretation of three‐dimensional anatomy during laparoscopic surgery.

### Strengths and limitations of exploring 3D anatomy

Participant comments not included in the formal analysis have highlighted additional advantages and areas for improvement in *Exploring 3D Anatomy*. In particular, participants noted the structural design of the course, deliberately included to reflect constructivist theoretical underpinnings, which enabled them to gradually construct their conceptualization of spatial anatomy: “[The course] *gradually built up, rather than throwing you in the deep end…if that Box Exercise [was] at the start, I would be a bit more confused*. ‘*I agree. I feel like the structure was really good. It built up using different methods to understand 3D spaces.”* If barriers to learner engagement described in the findings here can be overcome, additional limitations are likely to be similar to those of any online course. The absence of in‐person social interactions and video fatigue may result in participant attrition, and attempts were made to mitigate these factors in the original design of interactive and informal tasks within *Exploring 3D Anatomy*. To further address this area, there are several published guides and evidence‐based strategies that may be implemented to overcome such issues.^130^, ^131^, ^132^, ^133^

Like any online course, there are also potential unseen limitations relating to the monitoring and authenticity of student engagement and participation, and the effectiveness of tasks that are conducted remotely. However, as *Exploring 3D Anatomy* has been designed as a formative and optional supplement to formal anatomy education, learners who choose to use the course are likely to be motivated and engaged to follow the embedded principles and instructions. Course activities have been deliberately designed to be simple and accessible, and learners experiencing challenges in carrying out *Exploring 3D Anatomy* tasks are offered opportunities to engage in social learning by directly contacting educators for guidance and to interact with peers for support via the course discussion boards.

### Limitations of research study

While providing an understanding of learner experiences when using *Exploring 3D Anatomy*, the findings of this phenomenological interpretivist study could be strengthened by experimental and survey‐based investigations from a post‐positivist epistemological stance. While beyond the scope of the current study, future work may enable the large‐scale measurement of student performance and perceptions of their spatial awareness and three‐dimensional anatomical conceptualization, in order to identify if *Exploring 3D Anatomy* can enhance understanding, satisfaction, and confidence in novice anatomy learners. In doing so, data from learners who intend to apply to medicine, from those who are currently studying anatomy within other degree programs, or those at other higher education institutions, can also be collected in order to broaden the transferability of the findings. For example, *Exploring 3D Anatomy* is offered as an elective for medical students at the University of Cape Town, which provides an alternative learner population to investigate.

Additionally, although findings here demonstrate a spectrum or dichotomy of participant views, the convenience sampling approach used to recruit study participants did not explicitly address the potential for self‐selection from learners with greater interest in anatomy, art‐based activities, or remote learning. Future phenomenological studies may benefit from identifying participant backgrounds to ensure a range of views is included.

## CONCLUSION

*Exploring 3D Anatomy* is a free and accessible online course aimed at supporting spatial awareness in anatomy learners. Educators seeking to utilize *Exploring 3D Anatomy* with their own students, those wishing to develop their own supplementary online anatomy courses, those aiming to develop anatomy learning activities that focus on conceptual understanding rather than knowledge‐based learning, and those seeking to incorporate art‐based learning approaches into their teaching should all benefit from the detailed and comprehensive description of *Exploring 3D Anatomy* here, and from consideration of the findings of this phenomenological investigation. Specifically, educators will gain from an appreciation of different modes of inquiry favored by their students, which encompass their motivation to learn, varying perspectives on knowledge‐based or understanding‐driven dialogue and discussion, compartmental or conceptual approaches to cognition, and their level of experience.

## AUTHOR CONTRIBUTIONS

**Eleanor Gordon:** Writing – review and editing; conceptualization; resources; project administration; visualization. **Moksh Sachdeva:** Investigation; formal analysis; writing – review and editing. **Leonard Shapiro:** Conceptualization; project administration; visualization; formal analysis; validation; writing – review and editing; writing – original draft. **Jiayi Zhang:** Investigation; formal analysis; writing – review and editing. **Calum McCaig:** Investigation; formal analysis; writing – review and editing. **Iain D. Keenan:** Conceptualization; funding acquisition; writing – original draft; writing – review and editing; validation; methodology; formal analysis; project administration; supervision; data curation; resources.

## FUNDING INFORMATION

Newcastle University Faculty of Humanities and Social Sciences Institute of Creative Arts Practice, Newcastle University Careers Service, Newcastle University Faculty of Medical Sciences.

## Data Availability

The data that support the findings of this study are available on request from the corresponding author. The data are not publicly available due to privacy or ethical restrictions.
